# Optimizing Clinical Study Designs for CAR‐T Cell Therapy: Development of an Efficient Sampling Strategy Through Optimal Experimental Design

**DOI:** 10.1002/psp4.70274

**Published:** 2026-07-07

**Authors:** Fenja Klima, Anna M. Mc Laughlin, Robin Michelet, Wilhelm Huisinga, Katy Haussmann, Annette Künkele, Andrew C. Hooker, Charlotte Kloft

**Affiliations:** ^1^ Freie Universität Berlin Institute of Pharmacy, Department of Clinical Pharmacy and Biochemistry Berlin Germany; ^2^ Graduate Research Training Program PharMetrX Berlin, Potsdam Germany; ^3^ Pharmetheus AB Uppsala Sweden; ^4^ Institute of Mathematics University of Potsdam Potsdam Germany; ^5^ Berlin Center for Advanced Therapies Charité – Universitätsmedizin Berlin, Corporate Member of Freie Universität Berlin, Humboldt‐Universität zu Berlin, and Berlin Institute of Health Berlin Germany; ^6^ Department of Pediatric Oncology and Hematology Charité – Universitätsmedizin Berlin, Corporate Member of Freie Universität Berlin, Humboldt‐Universität zu Berlin, and Berlin Institute of Health Berlin Germany; ^7^ German Cancer Consortium (DKTK), partner Site Berlin and German Cancer Research Center (DKFZ) Heidelberg Germany; ^8^ Department of Pharmacy Uppsala University Uppsala Sweden

**Keywords:** cancer immunotherapy, CAR‐T cells, cellular therapy, clinical study design, modeling, optimal experimental design, simulation

## Abstract

CAR‐T cell therapy is a cellular cancer immunotherapy that has impressively improved outcomes in hematological malignancies compared to conventional treatments. Yet, many patients do not respond permanently. Nonlinear mixed‐effects modeling can support a better understanding of the unique and not fully understood dose‐exposure and exposure‐response relationships for CAR‐T cell therapy by integrating available knowledge into a quantitative, physiology‐motivated framework. However, to unfold its full potential, it requires informative and efficient study designs for clinical data collection. We aimed to develop an optimal experimental design framework informing a robust and feasible clinical CAR‐T cell study design based on a published mechanistic model of CAR‐T cell kinetics and tumor dynamics. By considering variability between study populations and parameter uncertainty, we (1) identified the minimal population size required to inform model parameters, (2) assessed different sampling strategies, and (3) informed flexible sampling windows instead of fixed sampling timepoints. The optimized study design consisted of 60 patients, three fixed assessments of tumor burden (days 0, 30 and 90), and three feasible CAR‐T cell sampling windows (days 2–4, 12–18 and 32–47 after infusion). In stochastic simulation and estimation, the optimized design with sampling windows showed better performance in informing model parameters, including those characterizing heterogeneous outcomes, than designs with fixed sampling timepoints, confirming its efficiency and robustness. This framework shall facilitate future feasible and resource‐efficient CAR‐T cell clinical data collection, thus showcasing the potential of optimal experimental design to advance the development and optimization of complex cancer immunotherapies in clinical trials and clinical practice.

## Study Highlights

**What Is the Current Knowledge on the Topic?**

- CAR‐T cell therapy is a cellular cancer immunotherapy with impressive efficacy, yet many patients eventually relapse. Nonlinear mixed‐effects modeling can help to better understand unique dose‐exposure‐response relationships. However, it relies on informative clinical data.

**What Question Did This Study Address?**

- This work aimed to inform a robust and feasible CAR‐T cell clinical study design considering patient number and sampling times. An optimal experimental design framework was developed using a published model of CAR‐T kinetics and tumor dynamics, considering variability between populations and parameter uncertainty.

**What Does This Study Add to Our Knowledge?**

- A design of 60 patients with three wide CAR‐T sampling windows and three fixed timepoints assessing tumor burden successfully informed model parameters, including those characterizing heterogenous outcomes. It outperformed scenarios with fixed sampling timepoints, illustrating its robustness and efficiency.

**How Might This Change Drug Discovery, Development, and/or Therapeutics?**

- This work demonstrated the potential of optimal experimental design to advance the development of complex cancer immunotherapies in clinical studies. The developed framework shall facilitate future efficient CAR‐T clinical data collection, contributing to leveraging its whole therapeutic potential.

## 1 Introduction

Chimeric antigen receptor‐T cell (CAR‐T) therapy is a genetically‐modified cellular cancer immunotherapy with impressive treatment outcomes in hematological indications and deep responses lasting for years in some patients [1, 2, 3, 4]. Currently, seven products are approved for hematological indications by the U.S. Food and Drug Administration and European Medicines Agency, and numerous are under investigation for hematological/solid tumors as well as autoimmune diseases [5, 6], moving CAR‐T therapy towards earlier lines of therapy and broader applications. However, while CAR‐T therapy has curative potential for some patients, up to 60% do not respond permanently [4].

Multiple factors have been associated with high variability in CAR‐T expansion, persistence, and treatment outcome [1, 7, 8], including biomarkers related to the disease (tumor type [7, 9], baseline tumor burden [10, 11, 12]), previous treatment (lines of therapy [7], lymphodepletion regimen [13]), CAR‐T product (phenotype and exhaustion status [10, 14, 15], ratio of CD4^+^/CD8^+^ cells [10, 12, 16]). Improving our quantitative understanding of the interplay between these factors and their impact on CAR‐T exposure and treatment outcome could support the advancement of CAR‐T product optimization and patient selection [8, 17, 18, 19]. Thus, to ultimately leverage the full potential of CAR‐T therapy, the collection of informative clinical data and its integration in quantitative frameworks is needed.

Nonlinear mixed‐effects (NLME) modeling allows to describe complex biological system dynamics, quantify layers of variability, and identify influential factors [20]. Moreover, it enables the integration of multi‐source and sparse data, leveraging all available information and rendering it a versatile methodology to approach questions related to CAR‐T kinetics, dynamics, and exposure‐response [20, 21]. While reducing the amount of data and resources is beneficial, precise and robust estimation of model parameters is essential to answer relevant questions. Ensuring sufficient information content when using sparse data thus requires carefully chosen study designs [22]. This is particularly important when data is scarce, as in CAR‐T therapy, where patient numbers and sample collection are constrained by rare indications, high treatment costs and the resource‐intensive bioanalysis required for CAR‐T phenotype quantification. In addition, the choice of sampling times is complex due to unique cellular kinetics and high interindividual variability (IIV). In such settings, it is crucial to maximize the informativeness of collected clinical data while considering the limits of available resources and practical feasibility.

Optimal experimental design (OED) enables efficient and informative study designs based on prior knowledge of the expected model structure, parameter values, and population characteristics [22, 23]. Moreover, it considers practical and ethical constraints such as feasible sampling windows and the maximal number of samples. Briefly, OED is based on an approximation of the expected Fisher Information Matrix (FIM) given a model of interest and a candidate design, from which expected relative standard errors (eRSEs) can be derived [22]. By using suitable design criteria as an objective function, such as D‐optimality, candidate designs can be optimized within a pre‐specified design space [22]. While no clinical data is required for an OED analysis, the optimized design is dependent on the model‐ and population‐related input. To consider uncertainty or variability in these input variables, various methods have been proposed and applied for developing robust designs [24, 25, 26]. OED combined with NLME modeling presents a powerful tool to inform clinical data collection a priori, enabling precise estimation of model parameters and ensuring an optimal use of available resources.

This work aimed to inform an efficient, robust, and feasible study design for clinical CAR‐T data collection capable of informing heterogeneous CAR‐T treatment outcomes, focusing on minimal population size and flexible sampling strategies by developing an OED framework using a published model of CAR‐T kinetics and tumor dynamics [12]. In doing so, it aimed to inform future clinical studies, thereby laying the foundation for a better mechanistic understanding of CAR‐T expansion, persistence, and their relationships with treatment outcomes to leverage the full potential of this therapy.

## 2 Methods

### 2.1 Model of CAR‐T Kinetics and Tumor Dynamics, and Virtual Patient Populations

An OED framework was developed based on a published NLME model [12] and virtual patient populations to inform a clinical CAR‐T study design enabling a better understanding of CAR‐T kinetics and tumor dynamics.

#### 2.1.1 Model Selection

Model selection was based on the following criteria: (1) a mechanistic model including both CAR‐T kinetic and tumor dynamic data; (2) estimation of IIV; (3) development based on clinical patient data.

**FIGURE 1:**
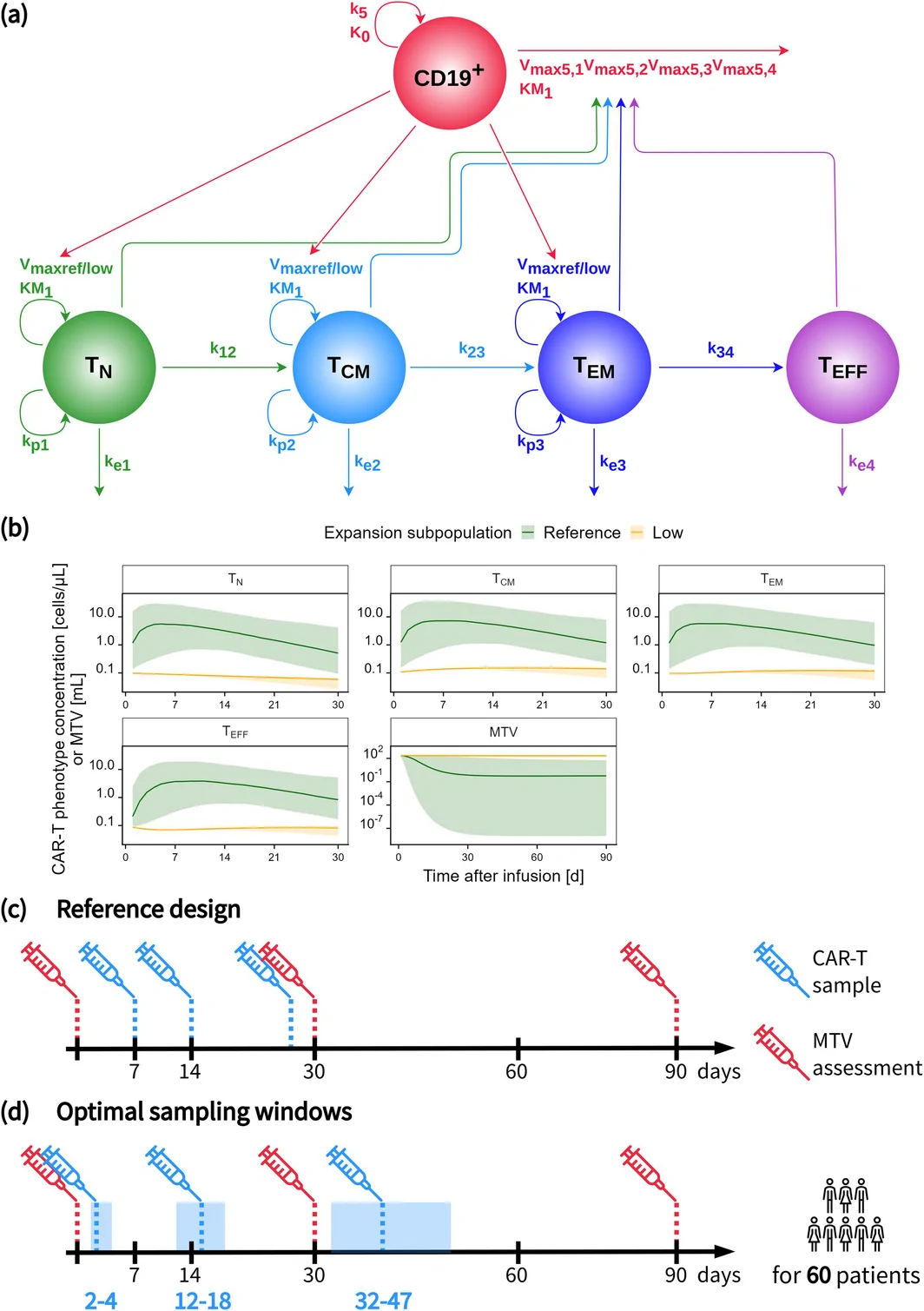
Schematic representation of the previously published model of CAR‐T kinetics and tumor dynamics adapted from Mueller‐Schoell et al. [12] (a), simulations illustrating variable CAR‐T expansion and tumor killing (b), the study design used as reference (c) and the developed optimal study design (d). (a) Homeostatic kinetics, tumor‐induced CAR‐T proliferation and CAR‐T‐induced tumor killing were described for five cellular species: CAR‐T phenotypes naïve (T_N_), central memory (T_CM_), effector memory (T_EM_), and effector (T_EFF_), and CD19^+^ metabolic tumor volume (CD19^+^). Homeostatic CAR‐T disposition followed first‐order processes: Differentiation of phenotypes with differentiation rate constants k_12_, k_23_, k_34_ (non‐red linear arrows pointing to the right); homeostatic proliferation with proliferation rate constants k_p1_, k_p2_, k_p3_, k_p4_ (non‐red circular arrows); cell death with death rate constants k_e1_, k_e2_, k_e3_, k_e4_ (non‐red arrows pointing downwards). Tumor‐induced CAR‐T proliferation was a nonlinear saturable process with maximum expansion rate per mL tumor volume (V_maxref_ and V_maxlow_) and concentration of T_N_, T_CM_ and T_EM_ at half‐maximum expansion (KM_1_). Proliferation of metabolic tumor volume (MTV) followed a logistic growth with proliferation rate constant (k_5_) and maximum tumor carrying capacity (K_0_). CAR‐T‐induced tumor killing was a nonlinear saturable process with maximum MTV killing rate by T_N_ (V_max5,1_), T_CM_ (V_max5,2_), T_EM_ (V_max5,3_), T_EFF_ (V_max5,4_) and MTV at half‐maximum killing rate (KM_5_). Adapted from Mueller‐Schoell et al. [12] licensed under CC BY 4.0 (http://creativecommons.org/licenses/by/4.0) (b) Concentrations of CAR‐T cell naïve (T_N_), central memory (T_CM_), effector memory (T_EM_), and effector (T_EFF_) phenotypes [cells/μL] and metabolic tumor volume (MTV) [mL] over time after CAR‐T infusion for two patients with reference (in green) or low (in orange) CAR‐T expansion. Stochastic simulations (*n* = 1000) considering interindividual variability were performed with an initial MTV of 229 mL. Solid lines represent the median simulated concentration and shaded areas simulated 80% prediction interval. (c) Sparse reference design for optimal design analysis. Blue symbols represent three CAR‐T sampling timepoints at days 7, 14, 28 after CAR‐T administration and red symbols represent three timepoints of metabolic tumor volume (MTV) assessment at days 0, 30, 90. (d) Optimized design with flexible sampling windows for three CAR‐T samples, three fixed MTV assessments and a minimal required population size of 60 patients. In addition to (b), blue shaded areas represent optimal sampling windows for each CAR‐T sample between days 2–4, 12–18 and 32–47.

The selected model was developed in our group based on clinical data from 19 patients with relapsed/refractory non‐Hodgkin lymphoma treated with axicabtagene ciloleucel [12]. CAR‐T pharmacokinetic (PK) samples from peripheral blood at days 7, 14 and 28 quantified via fluorescence‐activated cell sorting, and metabolic tumor volume (MTV) at baseline, months 1 and 3 assessed via positron emission tomography/computed tomography were used for parameter estimation. The model integrated the following aspects (Figure 1a): (1) CAR‐T disposition, i.e., differentiation, homeostatic proliferation and death of four CAR‐T phenotypes (naïve, central memory, effector memory, effector); (2) tumor‐induced nonlinear CAR‐T expansion with a reference and low expansion subpopulation captured by a mixture model on maximum CAR‐T expansion capacity (V_maxref_ and V_maxlow_); (3) tumor proliferation and CAR‐T‐induced tumor killing; (4) IIV on V_maxref_ and maximum MTV killing rate by central memory CAR‐T (V_max5,2_); (5) proportional residual unexplained variability (RUV) for each analyte [12]. To account for their exploratory nature due to the low patient number available for model development, covariates were removed from the model and model parameters were re‐estimated (Table 1). Importantly, the mixture model, providing two different sets of parameters to capture variable CAR‐T expansion, was implemented in the OED framework as dichotomous patient characteristic (i.e., reference or low expansion subpopulation, with subpopulation proportions equal to the published mixture probabilities), since the direct implementation of a mixture model was not supported. Figure 1b illustrates exemplary the variability in CAR‐T expansion and subsequent tumor killing between and within the subpopulations supported by the model of interest.

**TABLE 1:** Parameter estimates from adapted published nonlinear mixed‐effects model of CAR‐T kinetics and tumor dynamics used for optimal experimental design analysis. Covariate effects presented in the published model were removed. Adapted from Mueller‐Schoell et al. [12].

Parameter [unit]	Parameter description	Estimate (% CV)	RSE, %
**Fixed effects**			
V_maxref_ [(cells·μL^−1^)·day^−1^·mL^−1^]	Maximum expansion rate per mL tumor volume of T_N_, T_CM_, and T_EM_ for the reference (ref) and low expansion population	0.0166	40.2
V_maxlow_ [(cells·μL^−1^)·day^−1^·mL^−1^]		0.00067	33.8
KM_1_ [cells·μL^−1^]	T_N_, T_CM_, and T_EM_ concentration at half‐maximum expansion of T_N_, T_CM_, and T_EM_	1.07	30.3
k_p1_ [day^−1^]^a^	Homeostatic proliferation rate constant for T_N_	0.0005	fixed
k_p2_ [day^−1^]^a^	Homeostatic proliferation rate constant for T_CM_	0.007	fixed
k_p3_ [day^−1^]^a^	Homeostatic proliferation rate constant for T_EM_	0.007	fixed
k_12_ [day^−1^]	Differentiation rate constant from T_N_ to T_CM_	0.14	12.6
k_23_ [day^−1^]	Differentiation rate constant from T_CM_ to T_EM_	0.189	16.1
k_34_ [day^−1^]	Differentiation rate constant from T_EM_ to T_EFF_	0.356	18.8
k_e1_ [day^−1^]^b^	Death rate constant for T_N_	0.0102	17.9
k_e2_ [day^−1^]^b^	Death rate constant for T_CM_	0.0102	17.9
k_e3_ [day^−1^]^b^	Death rate constant for T_EM_	0.0102	17.9
k_e4_ [day^−1^]	Death rate constant for T_EFF_	0.513	17.9
V_max5,1_ [mL·day^−1^·(cells·μL^−1^)^−1^]^c^	Maximum killing rate of MTV by T_N_	2.73	37.9
V_max5,2_ [mL·day^−1^·(cells·μL^−1^)^−1^]	Maximum killing rate of MTV by T_CM_	4.28	37.9
V_max5,3_ [mL·day^−1^·(cells·μL^−1^)^−1^]^c^	Maximum killing rate of MTV by T_EM_	4.01	37.9
V_max5,4_ [mL·day^−1^·(cells·μL^−1^)^−1^]^c^	Maximum killing rate of MTV by T_EFF_	4.49	37.9
KM_5_ [mL]	MTV at half‐maximum killing rate	283	40.3
K_0_ [mL]^a^	Maximum tumor volume observable (tumor carrying capacity)	5000	fixed
k_5_ [day^−1^]^a^	Proliferation rate constant of MTV	0.0023	fixed
MIXP [−]	Estimated proportion of patients in the reference expansion population	85.1	9.94
**Random effects**			
IIV V_maxref_ ^d^	Interindividual variability in V_maxref_	2.08 (264.7%)	35.1
IIV V_max5,2_ ^d^	Interindividual variability in V_max5,2_	2.32 (302.9%)	37.5
RUV T_N_ ^e^	Residual unexplained variability of T_N_, T_CM_, T_EM_ and T_EFF_	0.301 (59.3%)	28.5
RUV T_CM_ ^e^		0.553 (85.9%)	27.2
RUV T_EM_ ^e^		0.903 (121.1%)	22.9
RUV T_EFF_ ^e^		0.394 (69.5%)	25.9
RUV CD19^+^ tumor^e^	Residual unexplained variability in observed metabolic tumor volumes	0.85 (115.7%)	35.8

Abbreviations: CV, coefficient of variation; IIV, interinidividual variability; MTV, metabolic tumor volume; RSE, relative standard error; RUV, residual unexplained variability.

^a^ Values were fixed as previously described by Mueller‐Schoell et al. [12].

^b^ Values were derived from k_e4_ as previously described by Mueller Schoell et al. [12].

^c^ Values were derived from V_max5,2_ as previously described by Mueller Schoell et al. [12].

^d^ Implemented as exponential relation, representing a lognormal parameter distribution. Estimates are reported as variance (%CV).

^e^ Implemented as additive relation on the log‐scale, i.e., exponential relation on the normal scale. Estimates are reported as variance (%CV).

#### 2.1.2 Virtual Patient Populations

For the OED analysis, individual initial MTV and CAR‐T expansion subpopulation (reference or low) were required as input. Virtual patient populations with varying initial MTV and individual CAR‐T expansion subpopulation were created for 10–150 patients. Initial MTV was simulated from a lognormal distribution (mean: $\log_{e}\left(229\;\mathrm{mL}\right)$, standard deviation: 1.47) truncated at a maximum tumor carrying capacity K_0_ of $\log_{e}\left(5000\;\mathrm{mL}\right)$. The distribution was informed by the MTV reported in the phase 3 trial ZUMA‐7, as the patient population in that study was similar but larger compared to the one used for model building [27]. The CAR‐T expansion subpopulation was simulated from a binomial distribution. The probability of belonging to the reference expansion subpopulation was informed by the proportion of patients with reference CAR‐T expansion reported by Mueller‐Schoell et al. i.e., 80% [12]. No correlation between initial MTV and CAR‐T expansion subpopulation was assumed. To account for variability between populations, 100 replicates were simulated for each population size.

### 2.2 Evaluation of Population Size and Sampling Strategies

The impact of population size and sampling strategies was assessed using design evaluations: Design variables were varied compared to a reference design (see below), and for each design, the expected FIMs were obtained for 100 population replicates. Resulting median eRSEs and interquartile ranges (IQRs) for each model parameter were summarized across population replicates and compared between designs. The reference design (Figure 1c) was the sparse sampling used for model development, i.e., three CAR‐T PK samples (days 7, 14, 28) and three assessments of MTV (baseline, days 30 and 90), commonly found in other clinical CAR‐T datasets as well [28, 29].

#### 2.2.1 Population Size

Patient numbers were varied from 10 to 150 patients with fixed reference CAR‐T and MTV sampling times. To identify the minimal population size required to inform all model parameters, a threshold of 50% median eRSE was applied as an acceptable threshold for parameter precision, commonly used in model evaluation [20].

#### 2.2.2 Sampling Strategies

To account for bioanalytical constraints when assessing different sampling times, i.e., uncertainty at very low concentrations, small fixed additive RUV parameters were added to the proportional RUV of the model, reducing the informativeness of timepoints with very low expected CAR‐T or MTV measurements [30]. To inform the magnitude of the additive RUV, no lower limits of quantification were available for CAR‐T phenotype or MTV measurements. Thus, it was assumed based on observed clinical data [12] and published variability thresholds [30]: Low plausible measurements for CAR‐T phenotypes and MTV were assumed to be 0.1 cells/μL and 0.5 mL with a variability of 50% CV, resulting in RUVs of 0.0025 (cells/μL)^2^ and 0.0625 mL^2^, respectively. A sensitivity analysis assessed the impact of different fixed additive RUVs on optimal sampling times (Supporting Information).

Using the model with additional fixed additive RUV and the identified minimally required population size, different sampling strategies were evaluated by adapting the reference sampling times for CAR‐T and/or MTV: (1) To explore the need for late sampling, the last CAR‐T or MTV sample or both were removed; (2) to assess the benefit of an additional CAR‐T sample during the important expansion phase, a fourth early CAR‐T sample was explored between days 0–13; (3) to assess the need for optimizing CAR‐T and MTV sampling timepoints, the last CAR‐T or MTV sample was shifted between days 18–90 or 30–180, respectively.

### 2.3 Design Optimization to Inform Sampling Windows

CAR‐T sampling windows were informed using D‐optimal design optimizations. The design space was based on the results of the evaluation of different population sizes and sampling strategies: For the minimal population size needed to inform all model parameters, derived in the previously described design evaluation, three CAR‐T PK samples were optimized between days 1–14, 7–28, and 14–60, while MTV assessments were fixed at baseline, days 30, and 90. To assess the impact of variability between populations and parameter uncertainty, design optimizations were carried out for (1) 100 population replicates and (2) 100 population replicates combined with parameter vectors sampled from the results of Sampling Importance Resampling (SIR) [31]. For each replicate, the resulting three sampling times were chronologically ordered, and for each CAR‐T PK sample, sampling windows were defined as the 5th‐95th percentile of the resulting 100 optimal timepoints. The efficiency of each optimized design was calculated as the normalized ratio of the D‐optimal criterion (FIM) before and after optimization (Equation (1)) [32]:

(1) $$\text{Efficiency}={\left(\frac{\left|\mathrm{FI}M_{\text{final}}\right|}{\left|\mathrm{FI}M_{\text{initial}}\right|}\right)}^{\frac{1}{n}}$$

where *n* is the number of estimated parameters. Efficiency was summarized across replicates to assess how informative the optimal design was compared to the reference design.

### 2.4 Stochastic Simulation and Estimation

The performance of the optimal design with sampling windows to inform model parameters was assessed using stochastic simulation and estimation (SSE), comparing three scenarios: (1) fixed reference sampling timepoints; (2) fixed optimal sampling timepoints, i.e., median optimized timepoints; (3) flexible optimal sampling windows. For scenarios with sampling windows, individual sampling timepoints were sampled from each window assuming a uniform distribution and no correlation between timepoints. SSE was performed with 500 replicates and considering parameter uncertainty. Population characteristics were simulated as described above for each replicate.

Relative estimation errors (REEs) were calculated for each model parameter (Equation (2)), summarized across replicates and compared between scenarios:

(2) $$\mathrm{REE}=\frac{{\text{estimate}}_{i,n}-{\text{true}}_{i,n}}{{\text{true}}_{i,n}}$$

with the estimated (estimate) and true (true) value of parameter *i* in replicate *n*.

### 2.5 Software and Optimal Design Settings

Data management, visualization and postprocessing of results were performed using R v4.4.0 with RStudio v2024.04.0. For OED analysis and model implementation, the R packages *PopED* v0.6.0 [22, 33] and *rxode2* v2.1.3 were used and the FIM was obtained using FO approximation.

SIR and SSE were performed in NONMEM v7.5.1 via PsN (Perl Speaks NONMEM) v4.7.0. SIR was performed with five iterations of samples (1000, 1000, 1000, 2000, 2000) and resamples (200, 400, 500, 1000, 1000) [31]. When considering uncertainty for the OED analysis and SSE, parameter vectors were obtained by sampling from SIR results. Code for OED and SSE model implementation is available on GitHub (https://github.com/Kloft‐Lab).

## 3 Results

### 3.1 Evaluation of Population Size and Sampling Strategies

#### 3.1.1 Population Size

**FIGURE 2:**
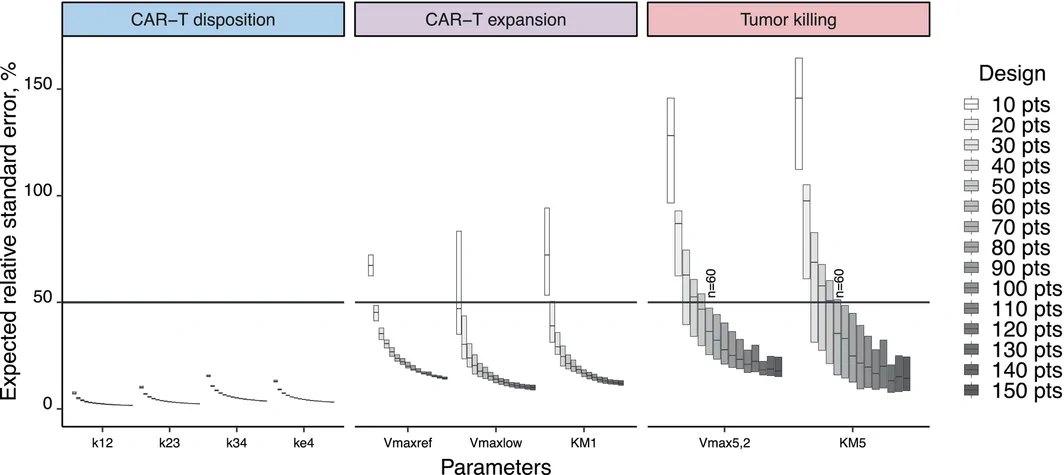
Expected relative standard errors (eRSE) comparing design evaluations of the reference design with 10–150 patients (step size: 10) for structural model parameters. For each design, 100 simulated population replicates were evaluated. Structural parameters are stratified by parameters related to homeostatic CAR‐T disposition (blue), tumor‐induced CAR‐T proliferation (violet) and CAR‐T‐induced tumor killing (red). Bars: Median eRSE and interquartile range (IQR) of replicates. Horizontal line: eRSE of 50% indicating sufficient parameter precision.

The assessment of population size revealed differences in expected parameter uncertainty between model parameters related to CAR‐T kinetics, tumor‐induced CAR‐T expansion and CAR‐T‐induced tumor killing (Figures 2 and S1): Applying a threshold of 50% median eRSE, 20 patients were sufficient to inform parameters related to CAR‐T disposition and CAR‐T expansion, while ≥ 60 patients were required to inform tumor‐killing‐related parameters. A further increase in population size above approximately 100 patients only had limited additional benefit in reducing eRSEs. However, larger patient populations led to reduced IQRs of eRSEs, corresponding to less impact of variability between populations.

#### 3.1.2 Sampling Strategies

**FIGURE 3:**
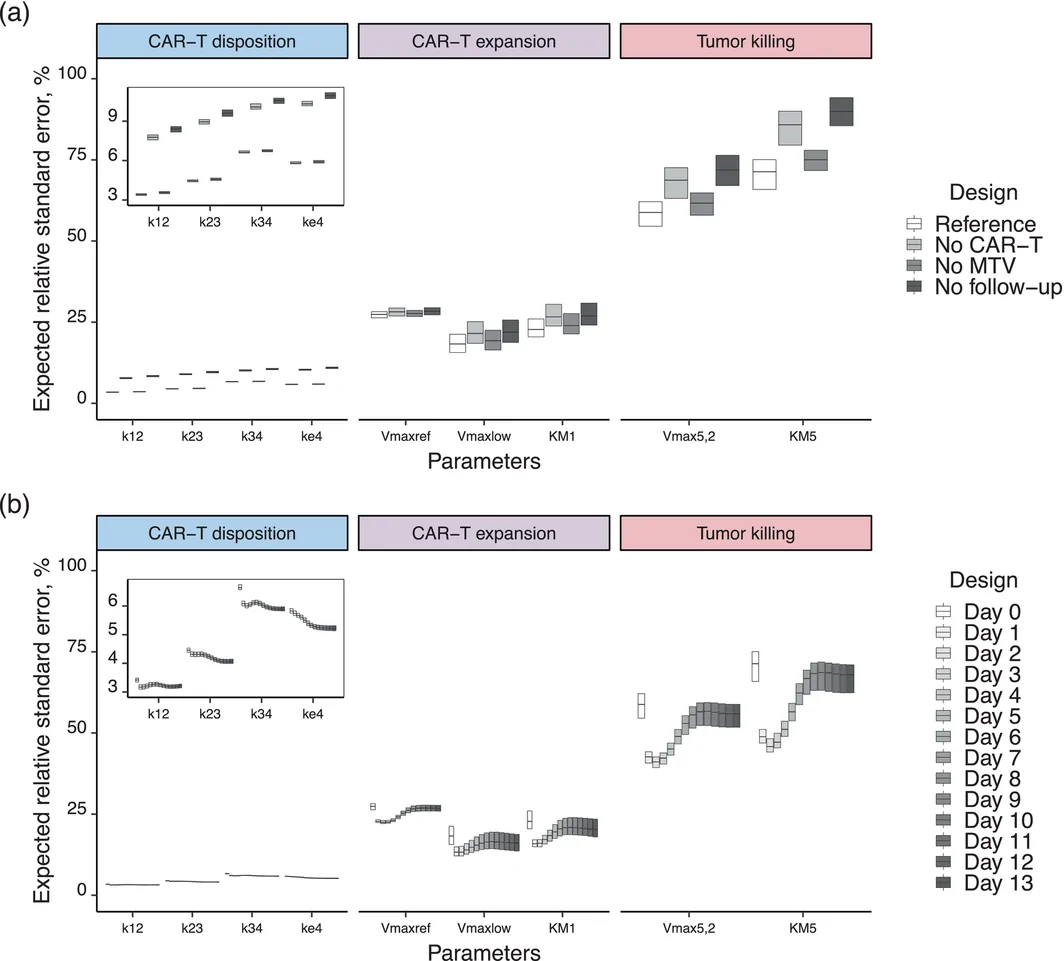
Expected relative standard errors (eRSE) comparing design evaluations of sampling strategies assessing the need for late or additional early samples for structural model parameters. For each design, 100 simulated population replicates were evaluated. Structural parameters are stratified by parameters related to homeostatic CAR‐T disposition (blue), tumor‐induced CAR‐T proliferation (violet) and CAR‐T‐induced tumor killing (red). For parameters related to CAR‐T disposition, a zoom into the relevant range of eRSEs is provided. Bars: Median eRSE and interquartile range (IQR) of replicates. (a) Compared to the reference scenario, the last CAR‐T sampling timepoint (No CAR‐T), the last assessment of metabolic tumor volume (No MTV) or both (No follow‐up) were removed. (b) A fourth CAR‐T sampling timepoint between days 0–13 after CAR‐T administration was added to the reference design.

**FIGURE 4:**
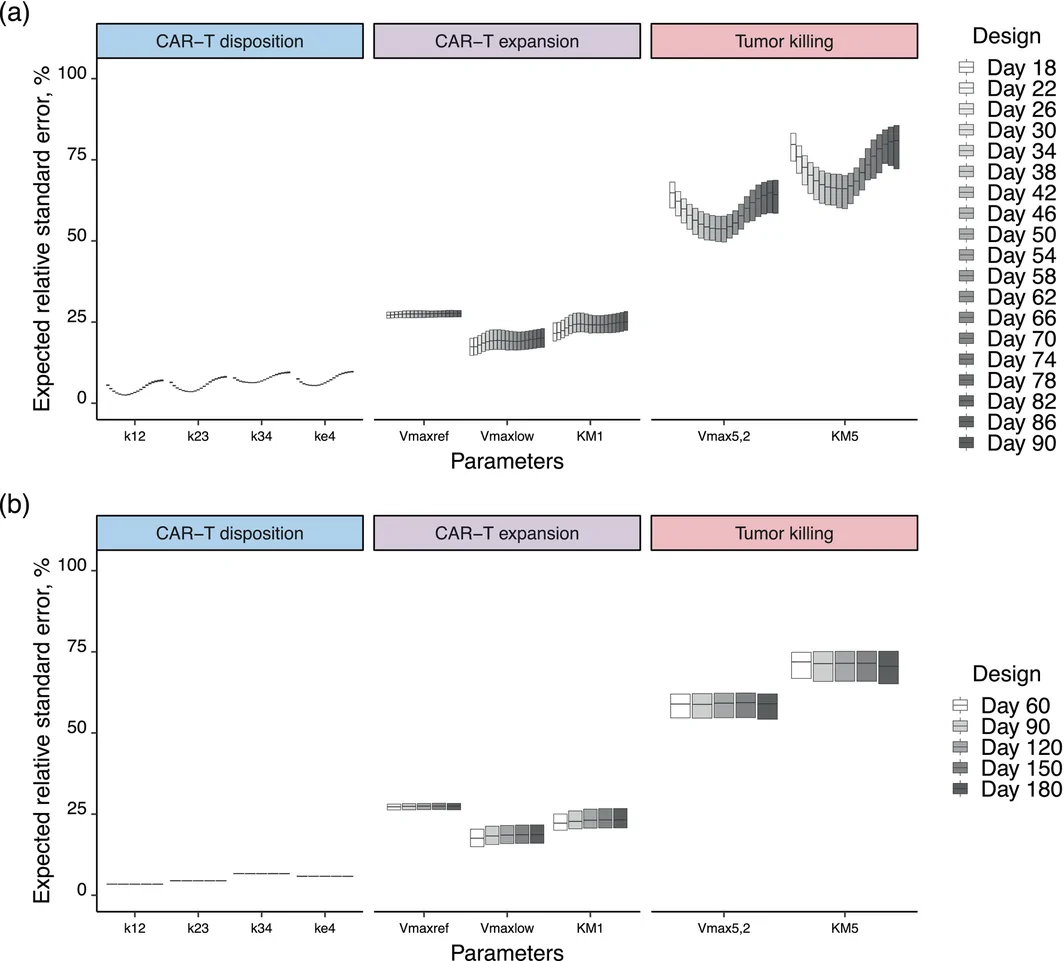
Expected relative standard errors (eRSE) comparing design evaluations of sampling strategies assessing the impact of shifting late sampling timepoints for structural model parameters. For each design, 100 simulated population replicates were evaluated. Structural parameters are stratified by parameters related to homeostatic CAR‐T disposition (blue), tumor‐induced CAR‐T proliferation (violet) and CAR‐T‐induced tumor killing (red). Bars: Median eRSE and interquartile range (IQR) of replicates. (a) Compared to the reference scenario, the last CAR‐T sampling timepoint was shifted between days 18–90. (b) Compared to the reference scenario, the last assessment of metabolic tumor volume was shifted between days 60–180.

Based on these findings, sampling strategies were evaluated for a population of 60 patients. A sensitivity analysis supported the extension of the model of interest with a small fixed additive RUV to account for bioanalytical constraints when assessing different sampling times, showing only limited impact of different RUVs on optimal sampling times (Supporting Information, Figures S2 and S3). Hence, the adapted model was applied for all evaluations of sampling strategies:

(1) Removing the last CAR‐T PK sample and/or MTV assessment led to an increase in eRSEs for all parameters (Figures 3a and S4a). The strongest increase of up to 18.6% points (1.3‐fold) was observed for parameters related to CAR‐T‐induced tumor killing in scenarios without late CAR‐T sampling, highlighting the need for late sampling timepoints especially for CAR‐T concentrations. (2) In contrast, the addition of an early CAR‐T sample led to an eRSE decrease of > 10% points in one or more parameters if taken between days 1–5 (Figures 3b and S4b). The strongest decrease of up to 25.5% points (0.6‐fold) was observed for parameters related to CAR‐T‐induced tumor killing at day 2. (3) Varying the last CAR‐T or MTV sampling timepoint revealed differences in the need for optimization of sampling times (Figures 4 and S5): Shifting the last CAR‐T sample impacted eRSEs for all model parameters. For instance, Figure 4a illustrates that the optimal sampling time for maximum MTV killing rate (V_max5,2_) would be day 50, while for maximum CAR‐T expansion capacity for both the low and reference expansion subpopulation (V_maxref_ and V_maxlow_) earlier timepoints, i.e., day 18, would be beneficial. In contrast, shifting the last MTV sample had only negligible impact on eRSEs across the wide range of days 60–180, corresponding to little need for optimization of MTV sampling timepoints (Figure 4b).

Taken together, assessments of sampling strategies demonstrated that model parameters could be informed with a minimal design consisting of three CAR‐T and MTV samples, including late samples, and that design optimization should focus on CAR‐T samples rather than MTV assessment.

### 3.2 Design Optimization to Inform Sampling Windows

To inform optimal sampling windows, the impact of variability between populations and parameter uncertainty on optimal sampling times was assessed. When considering variability between populations alone, 90% CIs of optimal sampling timepoints across replicates were very narrow, covering days 2–3, 13–15, and 38–39 for the first, second, and third CAR‐T sample, respectively. Thus, while crucial when evaluating population size, variability between populations had only a negligible impact on optimal sampling times. In contrast, when considering parameter uncertainty (in addition to variability between populations), 90% CIs of optimal sampling timepoints across replicates were considerably wider: They covered days 2–4 (median: 2), 12–18 (median: 15), and 32–47 (median: 39), highlighting the importance of considering parameter uncertainty when informing robust study designs.

The efficiency of optimized designs was similar in both cases with a median of 111% [90% CI: 108%–116%] when considering variability between populations alone and 111% [90% CI: 106%–118%] when additionally considering parameter uncertainty. This corresponds to 11% less patients required compared to the reference design to obtain the same parameter precision.

To ensure robust parameter estimation even with changing parameter values upon availability of new clinical CAR‐T data, optimal sampling windows were thus informed by design optimizations considering both parameter uncertainty and variability between populations. The final optimized design is summarized in Figure 1d, consisting of a population size of 60 patients, with three wide CAR‐T sampling windows and three fixed MTV sampling times at baseline, days 30 and 90.

### 3.3 Stochastic Simulation and Estimation

SSEs comparing scenarios with fixed sampling timepoints (reference and optimal) vs. flexible sampling windows demonstrated the suitability of sampling windows to inform model parameters. Across all scenarios, SSEs were robust: Out of 500 replicates, 96.8%–97.4% minimized successfully and passed common model evaluation criteria (no zero gradients reported for final parameter estimates; no estimates near pre‐specified boundaries). An additional 2.2%–3% of replicates did not minimize successfully only due to rounding errors and were included in the evaluation. With overall 99.4%–99.8% evaluable replicates across all scenarios, sampling windows showed the same robustness in successful parameter estimation as fixed sampling timepoints.

**FIGURE 5:**
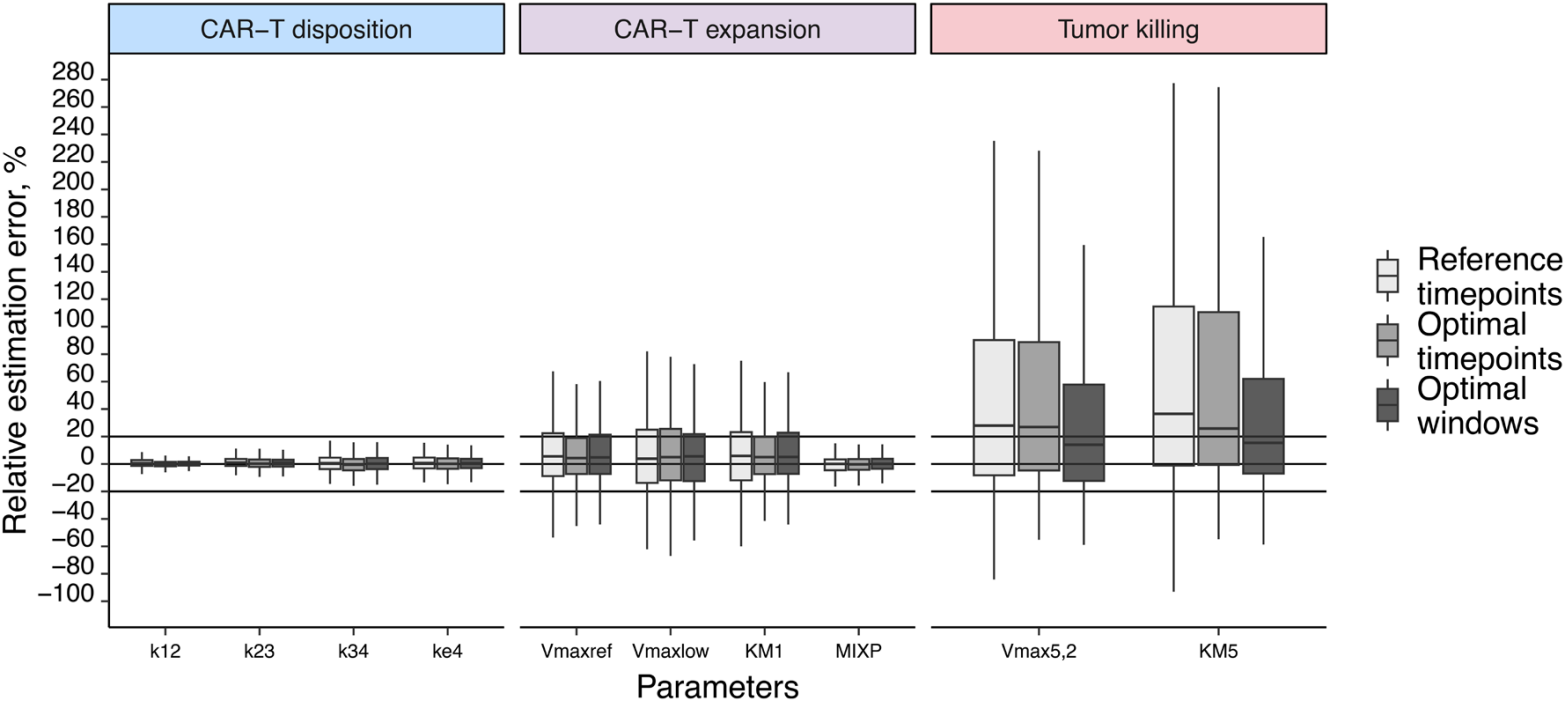
Relative estimation errors comparing the performance of three designs to inform structural model parameters. The tested designs all comprised 60 patients with three fixed assessments of metabolic tumor volume (days 0, 30, 90) and three CAR‐T sampling timepoints for each design as follows: (1) fixed timepoints from reference design (light gray), (2) fixed timepoints representing median of optimal sampling windows (medium gray), (3) timepoints sampled from optimal sampling windows for each patient individually applying uniform distribution. For each design, 500 replicates considering variability between populations and parameter uncertainty were evaluated. Structural parameters are stratified by parameters related to homeostatic CAR‐T disposition (blue), tumor‐induced CAR‐T proliferation (violet) and CAR‐T‐induced tumor killing (red). Boxplots: Median relative estimation errors and interquartile range (IQR). Whiskers: Largest/smallest value within a range of 1.5*IQR from the third/first percentile.

The evaluation of REEs revealed a similar or better performance of flexible sampling windows compared to both scenarios with fixed sampling timepoints (Figures 5 and S6). Importantly, differences between groups of parameters already observed during evaluations of population size and sampling strategies persisted in SSEs. Parameters related to CAR‐T disposition and CAR‐T expansion, including both sets of parameters related to reference or low CAR‐T expansion (V_maxref_ and V_maxlow_), were informed with high accuracy and precision, indicated by median REEs of close to zero (< 5.84%) and IQRs within or marginally extending the reference range of ±20% across all scenarios. In contrast, parameters related to CAR‐T‐induced tumor killing showed an overall lower accuracy and precision, but with noteworthy differences between scenarios with fixed sampling timepoints vs. sampling windows. For both scenarios with fixed sampling timepoints, median REEs were > 20%, ranging from 25.8%–36.5% with wide IQRs spanning 93.5% points–116% points, showing a clear trend towards positive REEs. Conversely, the scenario with flexible sampling windows achieved median REEs within ±20% and narrower IQRs spanning ≤ 70.1% points.

Of note, the study design with flexible sampling windows was the only design achieving median REEs within ±20% for all parameters, indicating the robustness and improved performance of sampling windows compared to fixed sampling timepoints. Interestingly, there were only minor differences between both scenarios with fixed sampling timepoints, showcasing that informing sampling windows based on uncertainty was more impactful than optimizing fixed sampling timepoints.

## 4 Discussion

In this work, we developed an OED framework based on a published model of CAR‐T kinetics and tumor dynamics to inform a robust and clinically feasible study design considering variability between populations and parameter uncertainty. With a minimal informative population size of 60 patients and wide sampling windows rather than fixed timepoints, the developed design facilitates timely and robust CAR‐T clinical data analysis, ultimately contributing to a better understanding of complex cancer immunotherapies.

To our knowledge, this is the first study applying OED to a cellular immunotherapy. Previously, OED has mostly been used in the context of pharmacometrics for conventional therapies, e.g., to derive informative sparse sampling strategies from rich designs or to inform early‐stage study design [34, 35, 36]. Minimal study design for conventional therapies like small molecules or monoclonal antibodies is facilitated by extensive experience with rich and sparse sampling strategies and a broad set of pharmacometric models developed in both settings. However, CAR‐T therapy represents a fundamentally different class of drugs: as a living drug product with unique cellular kinetics in the body, it exhibits distinct and still insufficiently understood dose‐exposure‐response relationships, and knowledge on optimal sampling strategies is limited [1, 37, 38, 39]. In recent years, multiple modeling approaches have been applied to CAR‐T therapy ranging from empirical population pharmacokinetics to quantitative systems pharmacology [8, 17, 18]: empirical piecewise‐linear models [40] provide adequate descriptive performance but are not suitable to provide mechanistic understandings. While mechanistic physiologically based pharmacokinetic and quantitative systems pharmacology models have been successful first steps in creating a quantitative framework to assess CAR‐T therapy, many rely on the availability of extensive pre‐clinical data, unrealistic to obtain in the clinical setting [41]. Different from previous OED analyses, we therefore based our design optimization on an already sparse reference design and aimed to increase its clinical flexibility and robustness to inform the model of interest.

To achieve an efficient design, we first informed a minimal population size required to inform all model parameters considering variability between populations. This yielded a population size of 60 patients as minimal threshold to inform a complex model of CAR‐T kinetics and tumor dynamics based on routine clinical data. Multiple pivotal clinical trials investigating new CAR‐T products demonstrated that such a population size is feasible to achieve despite rare indications [42, 43]. Moreover, several examples of prospective data collection during routine clinical practice have successfully achieved these numbers in single‐ or multicenter settings, highlighting the need for dedicated initiatives and collaboration [29, 44].

Based on these results, we evaluated different sampling strategies and aimed to inform flexible CAR‐T sampling windows rather than fixed timepoints by accounting for variability between populations and parameter uncertainty. To optimize sampling windows, the model was expanded with an additional fixed additive RUV, reducing the informativeness of very low concentrations. While allowing for intuitive understanding and simple implementation, this approach may impact the results of design optimizations compared to more complex strategies [30]. We therefore conducted a sensitivity analysis across a 200‐fold range of additive RUVs, which revealed no major impact on optimal sampling times, confirming the suitability for our analysis. The resulting sampling windows at days 2–4, 12–18, and 32–47 were biologically plausible, covering all important phases of the CAR‐T kinetic profile: a rapid onset of CAR‐T expansion, expected typical time of maximum CAR‐T concentration, and initial persistence up to 1 month after treatment, all of which have been associated with successful CAR‐T expansion, persistence, and favorable treatment outcomes [37, 42, 45, 46, 47, 48]. In contrast, timepoints of MTV assessment were not optimized, as the shift of the last MTV assessment between days 60–180 indicated no impact on OED results and the removal of the last MTV assessment alone resulted in only modestly worse OED results. A likely explanation is that tumor dynamics in the model, unlike CAR‐T kinetics, did not follow distinct kinetic phases. Thus, information content to inform these parameters was expected to be more evenly distributed over time and, consequently, to result in a reduced sensitivity of the model to the exact timing of MTV assessment. Moreover, most model parameters are primarily informed by data collected in the first month after treatment, as by day 60–180 a considerable proportion of CAR‐T cells already has been eliminated. Of note, the proposed design was a minimal design focused on clinical feasibility and minimization of resources, with three CAR‐T samples being sufficient to inform model parameters. However, the exploration of an additional early CAR‐T sample illustrated that the parameter precision could be further increased if resources for more samples are available.

The robust performance of the final design consisting of 60 patients, three CAR‐T sampling windows and three fixed MTV assessments was confirmed by an SSE. Of note, the assessment of sampling windows relied on the assumption that individual sampling timepoints were uniformly distributed and independent from each other. While plausible, the distribution of sampling timepoints in clinical practice may differ and should be evaluated using observational clinical data. Following this assumption, all model parameters were informed reasonably well, with similar or better accuracy and precision compared to scenarios with fixed sampling timepoints. This is an important step in OED, as eRSEs obtained from OED analysis represent the most optimistic minimal expected parameter uncertainty and the assessment of a design through stochastic simulations can highlight previously concealed challenges and pitfalls. The performed SSE confirmed that parameters related to tumor killing were most challenging to inform with sparse clinical data, possibly due to the nonlinear implementation and expected high correlations. Depending on the information content of future clinical data, this part of the model should therefore be revisited with special attention. Interestingly, both scenarios with fixed sampling timepoints behaved similarly in the tested setting across all model parameters with parameter uncertainty included in the simulations. This was in line with previous work showing that optimal designs obtained from incorrect underlying assumptions, e.g., model structure or parameter values, can be biased, supporting the use of sampling windows to increase clinical feasibility and robustness of study designs [24]. These results support the suitability of the proposed design for implementation in clinical practice.

While expected to provide a robust and flexible framework for clinical data collection, the results of the OED analysis are dependent on the underlying model and its scope needs to be considered: First, the model of interest did not consider the initial CAR‐T concentration decline after administration due to distribution. However, literature knowledge on CAR‐T biodistribution is sparse [19, 47] and models including the effect, e.g., as lag time, have estimated typical short durations of few hours to days [19, 40, 49]. While this simplification could affect the interpretation of the proposed first sampling window, a rapid onset of CAR‐T expansion might be an early marker for high CAR‐T exposure and favorable treatment outcomes [19, 49]. Second, our study did not include samples from the infusion product or later samples (> month 2) as they were not part of the reference design used for model development. The collection of such samples might inform aspects beyond the current model scope, such as the relation between CAR‐T product characteristics and CAR‐T kinetics or the characterization of longer‐term CAR‐T contraction and persistence [15]. To support early treatment decisions, it may however be more relevant to facilitate the identification of early predictors of CAR‐T treatment success after infusion: Multiple studies have linked CAR‐T exposure during the first month as a measure of expansion and short‐term persistence to favorable treatment outcomes [21, 42, 46, 47]. In that regard, the design proposed in this work successfully informed sets of parameters related to variable CAR‐T expansion and subsequent tumor killing (V_maxref_ and V_maxlow_). Thus, the proposed design is expected to provide a suitable foundation for further exploring varying CAR‐T treatment responses based on the model, by challenging its assumptions, validating its findings or expanding its scope by further characterizing CAR‐T expansion subpopulations.

The developed OED framework thus presents a promising setup for a pragmatic, yet informative clinical data collection, an important first step in accelerating our understanding of efficient clinical study designs for complex immunotherapies. This framework will likely support the validation and expansion of existing NLME models, e.g., by challenging and evaluating the model used in this work. As a potential future perspective, such a model could be applied in the context of adaptive design, e.g., to guide treatment decisions (switch to different treatment if CAR‐T cells are not expanding initially or upon tumor rebound) or to adapt sampling strategies for patients with expected long‐term CAR‐T persistence. Importantly, design elements may need to be adapted depending on the planned analysis and underlying model. For instance, other CAR‐T products and indications might be associated with slightly different cellular kinetics than the setting in which the model was developed [37, 39]. Since the robustness of the proposed design was successfully demonstrated considering parameter uncertainty, it would likely support a precise estimation of model parameters and cover relevant time points also for other CAR‐T constructs. In case of fundamentally different applications, for instance a different implementation of tumor burden, the robustness of the developed design can be further increased by incorporating different models in an OED analysis in case of uncertainty on the most adequate candidate model structure [26]. Moreover, for a highly regulated randomized controlled trial, flexible sampling windows might not be feasible, and biostatistical power‐calculations are required to determine the population size needed for a pre‐specified study power. However, the developed flexible OED framework can provide an additional perspective on multiple aspects of study design, including population size and the expansion to other models of interest, allowing a quantification of the impact of different sources of variability and a reduction of required resources [50]. It is important to note that—like model development—design optimization should be regarded as iterative process: As experience with CAR‐T and other immunotherapies grows and more data and models become available, knowledge on optimal sampling strategies needs to be refined to ensure maximal knowledge gain with minimal use of resources.

In conclusion, this analysis has demonstrated that OED is a powerful tool to inform study designs in the context of complex cancer immunotherapies. While study design for conventional therapies can rely on decades of experience, this is not the case for CAR‐T therapy, T‐cell engaging antibodies, or other examples of innovative treatment strategies [39]. Efficient and informative study designs are the foundation of successful drug development improving patient outcomes. Typically, clinical study designs are mandated by ethical, financial, and practical constraints. OED offers the potential to maximize the knowledge gained in these studies while minimizing required financial, human, or other resources.

This work provided a framework for OED analysis in the context of cancer immunotherapy, contributing to efficient and informative data collection and enabling a timely and meaningful model‐based analysis. In doing so, it paves the way for advancing the development of CAR‐T and other cancer immunotherapies in clinical studies, aiming to leverage their full potential.

## Author Contributions

All authors wrote the manuscript. F.K., A.K., A.C.H. and C.K. designed the research. F.K., A.M.M.L., R.M. and K.H. performed the research. F.K. analyzed the data.

## Funding

The authors have nothing to report.

## Disclosure

As an Associate Editor for CPT: Pharmacometrics & Systems Pharmacology, Andrew C. Hooker was not involved in the review or decision process for this paper.

## Conflicts of Interest

C.K. and W.H. report research grants from an industry consortium (AbbVie, AstraZeneca, Boehringer Ingelheim, F. Hoffmann‐La Roche, Merck KGaA, Novo Nordisk, Sanofi) for the graduate research training program PharMetrX and C.K. reports research grants from the H2020‐EU.3.1.3 (“FAIR”), all outside the submitted work. A.M.M. is an employee of Pharmetheus AB and a paid consultant to multiple pharmaceutical companies. All other authors declared no conflict interests for this work.

## Supporting information


**Figure S1:** Expected relative standard errors (eRSE) comparing design evaluations of the reference design with 10–150 patients for random effects model parameters. For each design, 100 simulated population replicates were evaluated. Parameters are stratified by interindividual variability and residual variability. Bars: Median eRSE and interquartile range (IQR) of replicates. Horizontal line: eRSE of 50% indicating sufficient precision.
**Figure S2:** Expected relative standard errors (eRSE) comparing design evaluations of sampling strategies with the last CAR‐T sampling timepoint shifted between days 18–90 when no additive RUV was added to the model. For each design, 100 simulated population replicates were evaluated. Structural parameters are stratified by parameters related to homeostatic CAR‐T disposition (blue), tumor‐induced CAR‐T proliferation (violet) and CAR‐T induced tumor killing (red). Bars: Median eRSE and interquartile range (IQR) of replicates.
**Figure S3:** Sensitivity analysis of the impact of additive residual variability (RUV) on optimal sampling times. Additive RUV was either the reference RUV (gray boxes), adapted only for CAR‐T observations (blue boxes) or for MTV observations (red boxes). Structural parameters are stratified by parameters related to homeostatic CAR‐T disposition (blue), tumor‐induced CAR‐T proliferation (violet) and CAR‐T induced tumor killing (red). Boxes: Sampling timepoint associated with minimal expected relative standard error (eRSE, central black bar in each box) and acceptable range of sampling timepoints resulting from a tolerance of 5% eRSE.
**Figure S4:** Expected relative standard errors (eRSE) comparing design evaluations of sampling strategies assessing the need for late or additional early samples random effects model parameters. For each design, 100 simulated population replicates were evaluated. Parameters are stratified by interindividual variability and residual variability. Bars: Median eRSE and interquartile range (IQR) of replicates. (a) Compared to the reference scenario, the last CAR‐T sampling timepoint (No CAR‐T), the last assessment of metabolic tumor volume (No MTV), or both (No follow‐up) were removed. (b) A fourth CAR‐T sampling timepoint between days 0–13 after CAR‐T administration was added to the reference design.
**Figure S5:** Expected relative standard errors (eRSE) comparing design evaluations of sampling strategies assessing the impact of shifting late sampling timepoints for random effects model parameters. For each design, 100 simulated population replicates were evaluated. Parameters are stratified by interindividual variability and residual variability. Bars: Median eRSE and interquartile range (IQR) of replicates. (a) Compared to the reference scenario, the last CAR‐T sampling timepoint was shifted between days 18–90. (b) Compared to the reference scenario, the last assessment of metabolic tumor volume was shifted between days 60–180.
**Figure S6:** Relative estimation errors comparing the performance of three designs to inform random effects model parameters. The tested designs all comprised 60 patients with three fixed assessments of metabolic tumor volume (days 0, 30, 90) and three CAR‐T sampling timepoints for each design as follows: (1) fixed timepoints from reference design (light gray), (2) fixed timepoints representing median of optimal sampling windows (medium gray), (3) timepoints sampled from optimal sampling windows for each patient individually applying uniform distribution. For each design, 500 replicates considering variability between populations and parameter uncertainty were evaluated. Parameters are stratified by interindividual variability and residual variability. Boxplots: Median relative estimation errors and interquartile range (IQR). Whiskers: Largest/smallest value within a range of 1.5*IQR from the third/first percentile.
