# Supplementary material for: Optimizing Clinical Study Designs for CAR‐T Cell Therapy: Development of an Efficient Sampling Strategy Through Optimal Experimental Design
Source: CPT Pharmacometrics Syst Pharmacol. 2026 Jul 7;15(7):e70274. doi: 10.1002/psp4.70274 (PMC13580764; doi:10.1002/psp4.70274)
Supplement: Supplementary file 1 [file PSP4-15-e70274-s001.docx]

**Supplementary Material**

**Supplementary Methods**

**Sensitivity analysis of additive RUV.** The model of interest was extended with an additional fixed additive residual unexplained variability (RUV) to prevent a biased selection of late sampling timepoints due to bioanalytical constraints, i.e., measurement uncertainty at very low concentrations. By introducing an additional additive RUV, the informativeness of timepoints with very low expected CAR‑T or MTV measurements was reduced. The expected impact of the magnitude of additional fixed additive RUV on the optimization of sampling timepoints was evaluated in a sensitivity analysis. The variance of additive RUV was varied in a 200-fold range from 0.00025-0.05 (cells/μL)^2^ for CAR-T phenotypes and 0.00625-1.25 mL^2^ for metabolic tumor volume (MTV) around the respective reference values of 0.0025 (cells/μL)^2^ and 0.0625 mL^2^. Next, the last CAR-T sample was shifted between days 18-90 for all scenarios with only proportional RUV or the adapted additive RUV. For each RUV value and model parameter, the timepoint associated with minimal median eRSE, i.e., optimal time point, and the acceptable range of sampling times allowing a tolerance of 5% eRSE compared to the minimum was identified and compared between scenarios.

**Supplementary Results**

**Sensitivity analysis of additive RUV.** To enable the identification of optimal sampling times while considering bioanalytical constraints, the inclusion of an additional small additive RUV was required: Using the original model with proportional RUV, the latest tested sampling time always achieved the lowest eRSEs, although bioanalytically implausible and with very little absolute change in eRSE (Supplementary Figure S2). A sensitivity analysis investigating the impact of different additive RUVs confirmed the suitability of the approach: Optimal sampling times obtained for the reference values of the additional additive RUV overlapped with the accepted range of sampling times of all other scenarios (Supplementary Figure S3). Of note, changing the RUV for CAR-T measurements resulted in a small shift of optimal sampling windows, whereas changing RUV for MTV assessment had no impact on optimal sampling windows, highlighting the importance of selecting adequate additive RUVs especially for CAR-T measurements.

**Supplementary Figures**

**Figure S1.** Expected relative standard errors (eRSE) comparing design evaluations of the reference design with 10-150 patients for random effects model parameters. For each design, 100 simulated population replicates were evaluated. Parameters are stratified by interindividual variability and residual variability. Bars: Median eRSE and interquartile range (IQR) of replicates. Horizontal line: eRSE of 50% indicating sufficient precision.

**Figure S2.** Expected relative standard errors (eRSE) comparing design evaluations of sampling strategies with the last CAR-T sampling timepoint shifted between days 18-90 when no additive RUV was added to the model. For each design, 100 simulated population replicates were evaluated. Structural parameters are stratified by parameters related to homeostatic CAR-T disposition (blue), tumor-induced CAR-T proliferation (violet) and CAR-T induced tumor killing (red). Bars: Median eRSE and interquartile range (IQR) of replicates.

**Figure S3.** Sensitivity analysis of the impact of additive residual variability (RUV) on optimal sampling times. Additive RUV was either the reference RUV (gray boxes), adapted only for CAR‑T observations (blue boxes) or for MTV observations (red boxes). Structural parameters are stratified by parameters related to homeostatic CAR-T disposition (blue), tumor-induced CAR-T proliferation (violet) and CAR-T induced tumor killing (red). Boxes: Sampling timepoint associated with minimal expected relative standard error (eRSE, central black bar in each box) and acceptable range of sampling timepoints resulting from a tolerance of 5% eRSE.

 **Figure S4.** Expected relative standard errors (eRSE) comparing design evaluations of sampling strategies assessing the need for late or additional early samples random effects model parameters. For each design, 100 simulated population replicates were evaluated. Parameters are stratified by interindividual variability and residual variability. Bars: Median eRSE and interquartile range (IQR) of replicates. (**a**) Compared to the reference scenario, the last CAR-T sampling timepoint (No CAR-T), the last assessment of metabolic tumor volume (No MTV), or both (No follow-up) were removed. (**b**) A fourth CAR-T sampling timepoint between days 0-13 after CAR-T administration was added to the reference design.

**Figure S5.** Expected relative standard errors (eRSE) comparing design evaluations of sampling strategies assessing the impact of shifting late sampling timepoints for random effects model parameters. For each design, 100 simulated population replicates were evaluated. Parameters are stratified by interindividual variability and residual variability. Bars: Median eRSE and interquartile range (IQR) of replicates. (**a**) Compared to the reference scenario, the last CAR-T sampling timepoint was shifted between days 18-90. (**b**) Compared to the reference scenario, the last assessment of metabolic tumor volume was shifted between days 60-180.

**Figure S6.** Relative estimation errors comparing the performance of three designs to inform random effects model parameters. The tested designs all comprised 60 patients with three fixed assessments of metabolic tumor volume (days 0, 30, 90) and three CAR-T sampling timepoints for each design as follows: (1) fixed timepoints from reference design (light gray), (2) fixed timepoints representing median of optimal sampling windows (medium gray), (3) timepoints sampled from optimal sampling windows for each patient individually applying uniform distribution. For each design, 500 replicates considering variability between populations and parameter uncertainty were evaluated. Parameters are stratified by interindividual variability and residual variability. Boxplots: Median relative estimation errors and interquartile range (IQR). Whiskers: Largest/smallest value within a range of 1.5*IQR from the third/first percentile.
